# Developing a predictive model for the efficacy of neoadjuvant chemoradiotherapy in locally advanced rectal cancer using multiparametric magnetic resonance imaging: An innovative approach

**DOI:** 10.1097/MD.0000000000046209

**Published:** 2026-05-12

**Authors:** Jing Cheng, Xinying Wu

**Affiliations:** a Department of Imaging, Nanjing First Hospital, Nanjing Medical University, Nanjing, Jiangsu, China; b Department of Imaging, Nanjing Hospital of Chinese Medicine Affiliated to Nanjing University of Chinese Medicine, Nanjing, Jiangsu, China; c Department of Radiology, Nanjing First Hospital, Nanjing Medical University, Nanjing, Jiangsu, China.

**Keywords:** attention mechanism, magnetic resonance imaging, neoadjuvant therapy, prediction model, rectal cancer

## Abstract

A predictive model was constructed based on traditional clinical magnetic resonance imaging (MRI) data to effectively predict the efficacy of neoadjuvant chemoradiotherapy (NCRT) for locally advanced rectal cancer (LARC). A retrospective analysis was conducted on the clinicopathological and imaging data of patients who underwent MRI prior to NCRT at Nanjing Traditional Chinese Medicine Hospital between April 2022 and June 2024. A total of 149 patients with histologically confirmed LARC were included. Based on the tumor regression grade (TRG) criteria for rectal cancer, patients were classified into TRG0 (n = 31), TRG1 (n = 34), TRG2 (n = 50), and TRG3 (n = 34). All patients underwent rectal MRI before treatment, and the imaging parameters (baseline status) were extracted. A predictive model based on multiparametric MRI indicators was developed to assess and predict the efficacy of neoadjuvant therapy in LARC. Statistically significant differences (*P*-values < .05) were observed in the imaging parameters, including the maximum sagittal tumor diameter, apparent diffusion coefficient (ADC), number and maximum diameter of lymph nodes, node stage, extramural vascular invasion, and circumferential resection margin (CRM), before and after treatment. A prediction model was constructed based on ablation experiments, and the best predictive performance was achieved when the feature combination included the maximum sagittal tumor diameter, ADC, maximum lymph node diameter, node stage, and CRM. The average classification accuracy, area under the curve, sensitivity, and specificity reached 87.53%, 0.85, 81.26%, and 79.32%, respectively. Among the top 50 ablation experiments ranked by predictive performance, the maximum sagittal tumor diameter, ADC, and node stage had higher proportions in the feature combinations. The prediction model constructed by using the optimized attention mechanism method has a high accuracy rate in predicting the therapeutic effect of LARC based on traditional clinical imaging data.

## 1. Introduction

Rectal cancer, a prevalent malignancy in the digestive system, has seen its incidence escalate globally over recent years, posing a grave threat to human life and health. In China, it ranks third among malignancies in terms of incidence and mortality rates for both males and females.^[1]^ Locally advanced rectal cancer (LARC) refers to rectal cancer where the tumor invades the muscularis propria, the epidural membrane of the rectum or adjacent organs, and/or is accompanied by multiple local lymph node metastases, with a certain risk of recurrence, where the choice of treatment strategy is crucial for patient prognosis.^[2]^ Traditional treatment modalities have shown limited efficacy in LARC, failing to guarantee patients’ quality of life. Consequently, the National Comprehensive Cancer Network’s clinical practice guidelines for rectal cancer recommend total mesorectal excision (TME) following neoadjuvant chemoradiotherapy (NCRT) as the standard of care for LARC patients.^[3]^ NCRT can shrink tumor size and downstage the clinical classification, thereby meeting patients’ needs for sphincter preservation and enhancing their quality of life. Furthermore, approximately 10 to 20% of rectal cancer patients achieve pathological complete response after NCRT.^[4]^ However, there is considerable variability in patients’ response to NCRT, with only 54 to 75% experiencing tumor downstaging. The mechanisms underlying this variability in treatment response remain unclear.^[5]^ Therefore, accurately predicting the efficacy of NCRT to optimize treatment strategies and avoid overtreatment or undertreatment is a pressing issue in the field of rectal cancer treatment.

Magnetic resonance imaging (MRI), as a noninvasive and high-resolution imaging modality, plays a pivotal role in the diagnosis, staging, stratification of different patient prognoses, and assessment of treatment response in rectal cancer.^[6]^ MRI not only furnishes morphological information, encompassing tumor size, peritumoral invasion, distant metastasis, and observation of treatment efficacy,^[7]^ but also delves into functional imaging at the molecular and cellular levels through techniques such as diffusion-weighted imaging (DWI)^[8]^ and dynamic contrast-enhanced imaging.^[9]^ These techniques provide microscopic insights into the tumor interior, including cell density and vascular distribution,^[10]^ which are of paramount importance for evaluating tumor biological behavior and predicting treatment response.

In recent years, with the rapid development of artificial intelligence and machine learning technologies, predictive models based on MRI imaging metrics have increasingly been applied in the assessment of tumor treatment efficacy. For instance, Lee et al^[11]^ utilized baseline T2-weighted MRI in combination with machine learning-based radiomics analysis to evaluate the tumor regression grade (TRG) in rectal cancer patients after NCRT. Jiang et al^[12]^ proposed a multimodal model based on preoperative MRI and deep learning to predict the therapeutic outcome of rectal cancer after NCRT. By extracting and analyzing quantitative features from MRI images and incorporating advanced algorithmic models, precise predictions of tumor treatment response can be achieved, providing robust support for clinical decision-making.^[13]^ Although some studies have focused on the prediction of the treatment efficacy of rectal cancer, there are few studies specifically targeting LARC, and the recurrence risk of patients at this stage is worth focusing on. There are significant individual differences in the efficacy of NCRT in LARC. Clinicians have limited effectiveness and subjective defects in evaluating the efficacy. Thus, Jiang et al^[14]^ established an radiomics model using pretreatment MRI multisequence image features and clinical parameters to predict the efficacy of NCRT in LARC patients. In addition, another study also used MRI-based radiomics to build predictive models, aiming to forecast good response in LARC patients before NCRT.^[15]^ While these model presented in the 2 study exhibits strong predictive capabilities, the radiomic features sourced from each imaging sequence for model construction lack sufficient clinical interpretability. As a result, we cannot determine the precise significance of individual features. It is worth noting that although traditional clinical imaging data holds significant clinical value and interpretability, it has not received sufficient attention. Traditional clinical imaging indicators are often more convenient and effective in guiding clinical decision-making. They provide clinicians with familiar and readily interpretable information that can directly influence treatment strategies. In contrast, the complex and opaque nature of radiomic features may limit their immediate utility in routine clinical practice. Therefore, constructing predictive models related to treatment response based on traditional clinical imaging features is of great importance for LARC patients. In view of this, this study aims to provide an efficient and convenient method for predicting the efficacy of LARC in clinical practice. We propose to construct an effective prediction model based on the traditional clinical MRI imaging data at baseline using the optimized attention mechanism method to assist clinical decision making.

## 2. Methods

### 2.1. Research framework

**Figure 1.**
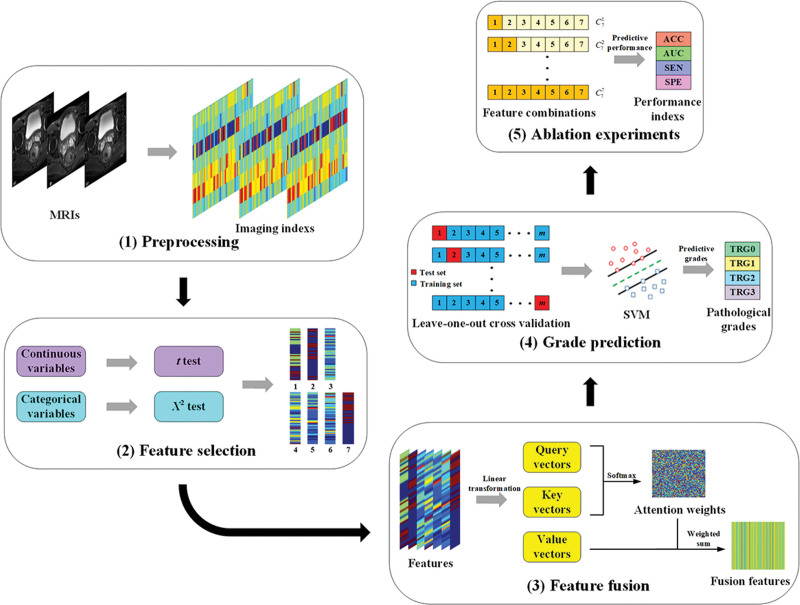
Research framework. (1) Preprocess the MRI of LARC to extract radiological indicators for each patient. (2) Employ the χ^2^ test for categorical variables and the paired *t*-test for continuous variables, using radiological indicators with statistical significance before and after treatment as features. (3) Leverage an attention mechanism for feature fusion, treating each feature as a value vector. Calculate the attention weights between key vectors and query vectors (learnable parameters), then perform weighted summation with the value vectors to obtain fused features. (4) Develop a support vector machine (SVM) prediction model, concurrently utilizing leave-one-out cross-validation to predict the efficacy of NCRT for LARC. (5) Conduct ablation experiments to compare the predictive performance of different feature combinations, exploring the predictive value of multiparametric MRI radiological models for the efficacy of NCRT in LARC. LARC = locally advanced rectal cancer, MRI = magnetic resonance imaging, NCRT = neoadjuvant chemoradiotherapy.

The research framework is illustrated in Figure 1.

### 2.2. Research subject

This study was approved by the Ethics Committee of Nanjing Traditional Chinese Medicine Hospital (Approval Number: KY2022186), and all patients provided written informed consent. All experiments were conducted in accordance with the criteria and regulations of the ARRIVE guidelines. We retrospectively collected data from rectal cancer patients diagnosed and treated at Nanjing Traditional Chinese Medicine Hospital between April 2022 and June 2024. A total of 149 patients were included, comprising 82 males and 67 females, with ages ranging from 28 to 68 years and a mean age of 60.86 ± 9.70 years. Inclusion criteria were as follows: histologically confirmed LARC; pelvic MRI and chest-abdomen-pelvis CT used for clinical tumor nodes metastasis staging prior to NCRT; all patients underwent TME surgery after NCRT; and complete MRI, clinical pathology data, and follow-up information before and after NCRT. Exclusion criteria included: familial adenomatous polyposis; prior history of other malignancies, abdominal or pelvic radiotherapy, or systemic chemotherapy related to malignancy; incomplete MRI examinations before or after NCRT with poor image quality or significant artifacts that precluded analysis; and incomplete clinical pathology data.

### 2.3. MRI examination

Using the Siemens Prisama 3.0T MR scanner equipped with an 18-channel phased-array surface coil encompassing the entire pelvic region, conventional MR scans were first conducted, following a sequence of axial T1-weighted imaging (T1WI), axial T2-weighted imaging (T2WI), sagittal T2WI, and coronal T2WI. Preparation of subjects prior to examination involved: scheduling the MRI for rectal cancer as far apart as possible from rectal endoscopic ultrasound and colonoscopy to prevent bowel irritation. Routine bowel preparation was not necessary for standard subjects. Subjects were instructed to fast from food and water for 4 hours before the scan, ensuring an empty stomach to prevent gastrointestinal reactions during contrast-enhanced scans. They were also advised to void urine and bowels before the scan; Intramuscular injection of 20 mg of anisodamine/scopolamine was administered half an hour before the examination to suppress motion artifacts caused by physiological movements of organs such as the gastrointestinal tract and bladder (patients’ contraindications should be confirmed before injection). For subjects undergoing contrast-enhanced scans, intravenous access was prepared beforehand, and subjects were instructed to practice breath-holding at end-expiration; and Routine safety checks were conducted on subjects before they entered the scanning room. Subject positioning involved: to minimize respiratory motion artifacts, it was recommended to use an elastic abdominal belt tightly wrapped around the subject’s torso to restrict pelvic respiratory movements; and The external landmark for centering was the midpoint of the line connecting both anterior superior iliac spines and the pubic symphysis.

Scanning protocols: For the 3-plane localization images, ensure a sufficiently large field of view (FOV) and scanning speed. Set 5 to 7 slices for sagittal localization, and 1 slice for both axial and coronal localization; For sagittal T2WI, place the localization line on the coronal localization image and adjust the vertical position of the localization box on the sagittal localization image. The inferior margin of the FOV should include the lowest part of the buttocks, while the superior margin should cover the L4 to L5 intervertebral space. Adjust the anterior-posterior position of the localization box to center the organ structures; For axial T2WI, set the localization line on the sagittal T2WI. Position the superior margin at the level of the L5 to S1 intervertebral space and the inferior margin to include the anus. Adjust the localization box on the axial localization image to center it on the body structure; For oblique axial T2WI, locate on the sagittal T2WI. Identify the tumor region and set the vertical scanning tangent plane along the long axis of the rectal lesion. Adjust the localization box on the axial localization image to center it on the body structure; For axial DWI, replicate the scanning range of axial T2WI but do not copy the slice thickness or FOV; For coronal T2WI, locate on the sagittal T2WI. The scanning range should cover the entire pelvic cavity, with the superior and inferior centers at the tip of the sacrum, the upper segment of the rectum, or the center of the acetabular roof. Adjust the localization box on the coronal localization image to center it on the body structure. Note that the FOV, slice thickness, and matrix must not be altered individually; any changes should be made uniformly to ensure a voxel size of 1.2 mm × 1.2 mm × 1.2 mm; and for axial T1WI, replicate the localization range of axial T2WI.

Gadolinium-based contrast agent (gadodiamide injection) is administered via a power injector at a rate of 2.5 mL/s. The scan sequences and parameters are shown in Table 1.

**Table 1:** MRI scan sequences and parameters for rectal cancer.

Scan sequences	Repetition time (TR), ms	Echo time (TE), ms	Resolution	FOV (mm × mm)	Layer thickness, mm	Layer spacing, mm
Axis T1WI	636	10	256 × 320	220 × 220	3	0.6
Axis T2WI	5870	105	288 × 320	220 × 220	3	0.6
Sagittal T2WI	4500	94	288 × 320	220 × 220	3	0.3
Coronal T2WI	4540	94	288 × 320	220 × 220	3	0.3
Axis DWI (*b* = 50, 800 s/mm^2^)	3600	50	102 × 134	320 × 320	4.5	1.35
Axial enhancement T1WI	3.48	1.3	182 × 320	320 × 320	3	0.6
Sagittal enhancement T1WI	3.8	1.41	224 × 320	280 × 280	3	0.6
Coronal enhancement T1WI	3.67	1.34	224 × 320	320 × 320	3	0.6

DWI = diffusion-weighted imaging, FOV = field of view, MRI = magnetic resonance imaging, T1WI = T1-weighted imaging, T2WI = T2-weighted imaging.

### 2.4. Image preprocessing

Two junior radiologists (radiologist 1 and radiologist 2) with 5 years of imaging diagnosis experience were trained, and then they independently read the images without knowing the postoperative pathology of the patients, analyzed the tumor location, tumor nodes metastasis stage, circumferential resection margin (CRM), extramural vascular invasion (EMVI), etc. Tumor segmentation was performed using the open-source software ITK-SNAP (https://itk.org/). The apparent diffusion coefficient (ADC) images of the patients were imported. A physician with 2 years of experience in imaging diagnosis manually delineated the region of interest layer by layer on the ADC images. To improve the accuracy during the measurement process, other sequence images of the patients, such as T2WI, T1WI, and DWI, were referred to. The selection of region of interest includes the tumor as a whole, and during the delineation process, intestinal contents and peripheral blood vessels should be avoided. During the delineation process, another physician with 5 years of experience in imaging diagnosis provided guidance to review the segmented images. For patients with objections, consensus was reached through discussion.

### 2.5. Pathological grading

**Figure 2.**
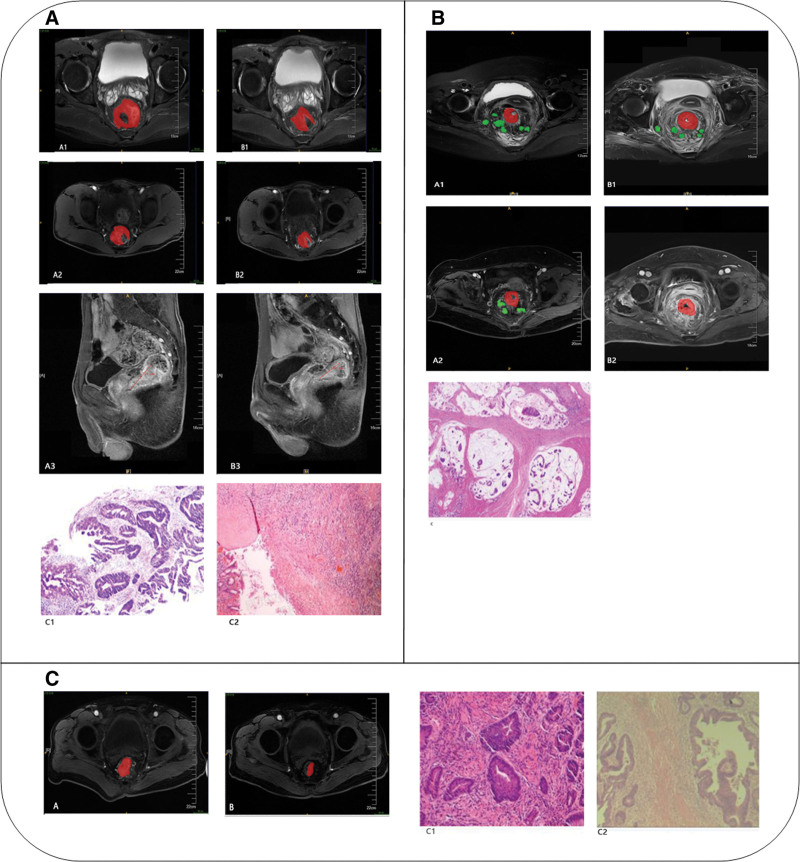
Typical cases. (A) A 50-yr-old male patient with rectal cancer. Pre-neoadjuvant therapy images (A1–A3) and post-neoadjuvant therapy images (B1–B3) include axial plain, axial enhanced, and sagittal enhanced MR scans, respectively. A significant reduction in the tumor size was observed post-therapy, accompanied by localized edema in the intestinal wall. Pathological diagnosis (C1): Moderately differentiated rectal adenocarcinoma. Pathological staging (C2): ypT0N0MX (yp = post-treatment pathological staging following neoadjuvant therapy). TRG: TRG0. (B) A 69-yr-old female patient with rectal cancer. Pre-neoadjuvant therapy images (A1–A2) show plain and enhanced scans, respectively, while post-neoadjuvant therapy images (B1–B2) demonstrate similar scans. Prior to treatment, the tumor circumferentially involved the bowel wall with multiple enlarged lymph nodes surrounding it, and a CRM+. Post-treatment, there was no apparent reduction in tumor size, with persistent enlarged lymph nodes, intestinal wall edema with necrosis, and CRM + status. Pathological diagnosis (C): Moderately to poorly differentiated rectal adenocarcinoma. Pathological staging: yPT3N2aMx (yp = post-treatment pathological staging following neoadjuvant therapy). TRG: TRG2. (C) A 69-yr-old male patient with rectal cancer. Pre-neoadjuvant therapy MR enhancement (A) shows a tumor occupying approximately 3/4 of the circumference of the bowel wall with small lymph nodes and vascular shadows surrounding it. Post-neoadjuvant therapy MR enhancement (B) reveals a tumor occupying about 2/3 of the circumference. Pathological diagnosis (C1): Moderately differentiated rectal adenocarcinoma. Pathological staging (C2): ypT3N0MX (yp = post-treatment therapy pathological staging following neoadjuvant therapy). TRG: TRG3. The red areas represent the lesion and the green areas represent the lymph nodes. CRM = circumferential resection margin, MR = magnetic resonance, TRG = tumor regression grade.

The postoperative specimens were processed and reviewed by senior pathologists. Based on the colorectal cancer staging system published by the American Joint Committee On Cancer in collaboration with the Union for International Cancer Control in 2018, patients were classified into a complete response group and a non-complete response group, depending on the absence or presence of adenocarcinoma cells in the pathological outcomes. With reference to the American Joint Committee on Cancer criteria in pathology, the TRG for rectal cancer was categorized as follows: TRG0 (Fig. 2A) indicating no residual cancer cells (complete response); TRG1, isolated single cancer cells or small clusters of cancer cells (nearly complete response); TRG2 (Fig. 2B), residual tumor cells present (minimal response); and TRG3 (Fig. 2C), minimal or no tumoricidal effect (poor response).

### 2.6. Prediction model construction

The objective of this study is to extract key clinical parameters of the baseline status as features from rectal cancer patients, employ an attention mechanism for feature fusion, and construct an support vector machine (SVM) model to achieve accurate assessment and prediction of the therapeutic effect after neoadjuvant therapy for rectal cancer (based on TRG grading results).

Data were collected from the clinical records of rectal cancer patients, with parameters such as N stage, EMVI, maximum tumor diameter, ADC value, number of lymph nodes, maximum lymph node diameter, and CRM status selected as features. Data preprocessing involved standardization or normalization methods to ensure comparability among different features in numerical terms.^[16]^ The standardization formula utilized is as follows:


$$\begin{array}{l} z=\frac{\left(x-\mu \right)}{\sigma }. \end{array}$$ (1)

where × represents the raw data, *μ* denotes the mean, *σ* signifies the standard deviation, and *z* stands for the standardized data.

The core concept of the attention mechanism lies in calculating the contribution (weight) of each feature towards the final prediction outcome and subsequently summing these weighted features.^[17]^ To achieve this, a neural network layer based on the attention mechanism is designed for the fusion of multiple features.^[18]^ The formula for computing the attention weights is as follows:


$$\begin{array}{l} Attention(q,k,v)=softmax(\frac{qk^{T}}{\sqrt{d_{k}}})v. \end{array}$$ (2)

where *q* represents the query vector, *k* denotes the key vector, and *v* signifies the value vector. *d*_*k*_ is the dimension of the key vector, and the softmax function is employed to normalize the weights. In our study, we treat each feature as a value vector and compute the attention weights between the key vectors and learnable query vectors. These weights are then used to perform a weighted summation of the value vectors, yielding the fused features.

To construct the SVM classifier, the fused features are fed as input. A Gaussian kernel function is utilized to map the input features into a high-dimensional space, where an optimal hyperplane is sought to differentiate samples of different classes.^[19]^ The objective function of the SVM is formulated as:


$$\begin{array}{l} \underset{w,b,\xi }{\min}\;\frac{1}{2}{∥w∥}^{2}+C\sum_{i-1}^{N}\xi_{i}. \end{array}$$ (3)

The constraints are as follows:


$$\begin{array}{l} y_{i}(w\cdot \phi (x_{i})+b)\geq 1-\xi_{i}\xi_{i}\geq 0i=12...N. \end{array}$$ (4)

where ***w*** denotes the weight vector, *b* represents the bias term, *ϕ*(**x**_*i*_) is the kernel function that maps input features into a high-dimensional space, *ξ*_*i*_ stands for the slack variable, and *C* is the regularization parameter.

Model validation was conducted using the leave-one-out cross-validation method.^[20]^ In each iteration, 1 sample was designated as the test set, with the remaining samples serving as the training set, to evaluate the model’s performance on an independent test set. This approach ensures that every sample is used as a test set once, providing a comprehensive assessment of the model’s performance.

As predictive performance indicators, classification accuracy (ACC), specificity (SPE), sensitivity (SEN), and area under the curve (AUC) were employed.^[21]^ The calculation of these performance metrics is as follows:


$$\begin{array}{l} ACC=\frac{TP+TN}{TP+TN+FP+FN}. \end{array}$$ (5)

$$\begin{array}{l} SPE=\frac{TN}{TN+FP}. \end{array}$$ (6)

$$\begin{array}{l} SEN=\frac{TP}{TP+FN}. \end{array}$$ (7)

$$\begin{array}{l} AUC=P(P_{Positive}>P_{Negative}). \end{array}$$ (8)

where TP stands for true positives (the number of samples correctly predicted as positive), TN for true negatives (the number of samples correctly predicted as negative), FP for false positives (the number of samples incorrectly predicted as positive), and FN for false negatives (the number of samples incorrectly predicted as negative). AUC measures the probability that a classifier ranks a randomly chosen positive sample higher than a randomly chosen negative sample.

Given that a larger number of parameters does not necessarily equate to better performance, as excess parameters may lead to overfitting and diminish the model’s generalization ability,^[22]^ we selected 7 statistically significant parameters before and after treatment: maximum tumor diameter in the sagittal plane, ADC value, number of lymph nodes, maximum lymph node diameter, N stage, EMVI, and CRM. These were designated as feature 1, 2, 3, 4, 5, 6, and 7, respectively, and used as inputs for the prediction model. Ablation experiments^[23]^ were conducted, totaling 127 experiments (mathematical combinatorial expression: $C_{7}^{1}+C_{7}^{2}+C_{7}^{3}+C_{7}^{4}+C_{7}^{5}+C_{7}^{6}+C_{7}^{7}=127$).

## 3. Statistical analysis

The statistical analysis of the data was conducted using SPSS version 25.0 software. For categorical variables, including N stage, T stage, EMVI, and CRM, a paired chi-square test was employed. For continuous variables, such as tumor circumferential ratio, maximum tumor diameter in the sagittal plane, ADC value, distance from the tumor to the anus, number of lymph nodes, and maximum lymph node diameter, a paired *t*-test was utilized.^[24]^ A *P*-value of <.05 was considered statistically significant, indicating a difference that was not due to chance.

## 4. Results

### 4.1. Participants inclusion

Ultimately, our study included a total of 149 patients, among whom 82 were male and 67 were female. There were 31 cases of TRG0, 34 cases of TRG1, 50 cases of TRG2, and 34 cases of TRG3.

### 4.2. Comparison of image parameters

**Figure 3.**
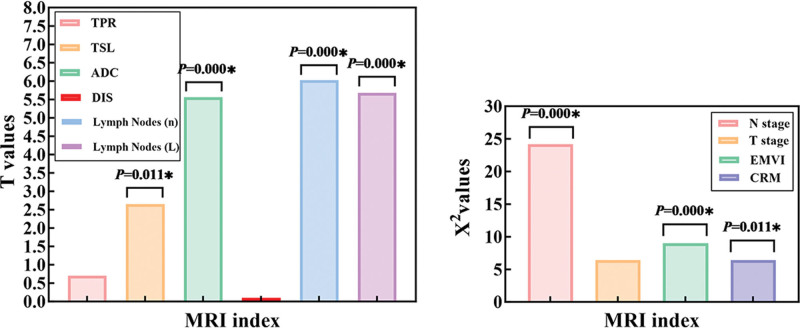
Statistical results. Before and after treatment, no statistically significant differences were noted in the tumor circumferential ratio, distance from the tumor to the anus, and T stage among patients (all *P*-values > .05). However, statistically significant differences were observed in the maximum tumor diameter in the sagittal plane, ADC value, number of lymph nodes, maximum lymph node diameter, N stage, EMVI, and CRM, with *P*-values of .011, .000, .000, .000, .000, .003, and .011, respectively. ADC = apparent diffusion coefficient, CRM = circumferential resection margin, EMVI = extramural vascular invasion.

Before and after treatment, no statistically significant differences were noted in the tumor circumferential ratio, distance from the tumor to the anus, and T stage among patients (all *P*-values > .05). However, statistically significant differences were observed in the maximum tumor diameter in the sagittal plane, ADC value, number of lymph nodes, maximum lymph node diameter, N stage, EMVI, and CRM, with *P*-values of.011, .000, .000, .000, .000, .003, and .011, respectively. The specific results are presented in Tables 2 and 3, as well as Figure 3. These findings suggest that the maximum tumor diameter in the sagittal plane, ADC value, number of lymph nodes, maximum lymph node diameter, N stage, EMVI, and CRM may serve as effective imaging biomarkers for assessing the efficacy of NCRT.

**Table 2:** *t*-Test of samples before and after NCRT.

Items	Average/frequency	Standard deviation	*t*	*P*
Tumor circumferential ratio†	0.760	0.217	0.701	.487
Tumor circumferential ratio‡	0.740	0.227		
Tumor sagittal the longest diameter†	53.980	19.549	2.648	.011*
Tumor sagittal the longest diameter‡	46.000	19.133		
ADC value†	0.970	0.251	5.562	.000*
ADC value‡	1.223	0.292		
Distance from tumor to anus†	39.370	13.368	0.10	.992
Distance from tumor to anus‡	39.350	14.082		
Lymph node number†	4.040	3.385	6.030	.000*
Lymph node number‡	1.180	1.537		
Maximal lymph node diameter†	6.270	4.974	5.679	.000*
Maximal lymph node diameter‡	2.480	3.723		

ADC = apparent diffusion coefficient, NCRT = neoadjuvant chemoradiotherapy.

* Statistically significant differences between groups, with *P* < .05.

† Before NCRT.

‡ After NCRT.

**Table 3:** Chi-square test of samples before and after NCRT.

Items	Average/frequency	χ^2^	*P*
N stage†	10/20/19	24.170	.000*
N stage‡	33/14/2		
T stage†	4/43/2	6.397	.094
T stage‡	14/34/1		
EMVI†	35/14	9.000	.003*
EMVI‡	44/5		
CRM†	31/18	6.400	.011*
CRM‡	39/10		

CRM = circumferential resection margin, EMVI = extramural vascular invasion, NCRT = neoadjuvant chemoradiotherapy.

* Statistically significant differences between groups, with *P* < .05.

† Before NCRT.

‡ After NCRT.

### 4.3. Prediction model results

The ablation experiment results revealed that the optimal predictive performance was achieved when the feature combination comprised the maximum sagittal diameter of the tumor, ADC value, maximum diameter of lymph nodes, N stage, and CRM, with ACC, AUC, SEN, and SPE reaching 87.53%, 0.85, 81.26%, and 79.32%, respectively.

The specific ablation experiment results for the 7 trials with better predictive outcomes using combinations of 1 to 7 parameters are presented in Table 4. The findings indicated that, while the ADC value (Feature 2) exhibited the highest predictive power when used as a single feature input, its ACC was only 67.18 ± 2.94%, suggesting a relatively weak predictive capability of the model when relying solely on the ADC value. The AUC value was 0.63 ± 0.04, which falls within the lower range between 0.5 and 1, indicating a less robust discriminatory ability of the model. Additionally, its SEN and SPE were also relatively low, at 59.61 ± 2.07% and 68.74 ± 2.84%, respectively. When the N stage (Feature 5) was combined with the ADC value (Feature 2), there was a significant improvement in the model’s ACC, AUC, SEN, and SPE, highlighting the N stage as an important predictor. With the further inclusion of the maximum sagittal diameter of the tumor (Feature 1) and the number of lymph nodes (Feature 3) to the combination (1 + 2 + 3 + 5), the model’s performance continued to enhance, indicating that these 3 features also possessed high predictive value. When the feature combination reached 1 + 2 + 4 + 5 + 7 (encompassing the maximum diameter of lymph nodes and CRM), the model’s accuracy attained an optimal level of 87.53 ± 0.68%, with an AUC value of 0.85 ± 0.03, which represented a relatively high standard. This suggested that these combinations of features could effectively predict the tumor status. However, when the EMVI (Feature 6) was added to form the complete feature set (1 + 2 + 3 + 4 + 5 + 6 + 7), the model’s performance did not further improve but slightly declined, potentially due to redundancy or interaction among the features. The predictive results revealed that the ADC value and N stage were 2 crucial predictors, capable of significantly enhancing the model’s predictive capability when used alone or in combination. The maximum sagittal diameter of the tumor, the number of lymph nodes, the maximum diameter of lymph nodes, and CRM were also predictive features, but their contributions might vary based on specific scenarios.

**Table 4:** Prediction results.

Feature number	ACC	AUC	SEN	SPE
2	67.18 ± 2.94%	0.63 ± 0.04	59.61 ± 2.07%	68.74 ± 2.84%
2 + 5	79.76 ± 0.31%	0.78 ± 0.02	72.58 ± 0.37%	71.94 ± 2.61%
1 + 2 + 3	79.80 ± 3.58%	0.79 ± 0.03	71.36 ± 1.52%	72.83 ± 0.49%
1 + 2 + 3 + 5	85.66 ± 1.84%	0.81 ± 0.01	81.41 ± 3.22%	79.17 ± 1.26%
1 + 2 + 4 + 5 + 7	**87.53** ± **0.68%**	**0.85 ± 0.03**	81.26 ± 0.12%	**79.32** ± **1.37%**
1 + 2 + 3 + 4 + 5 + 7	85.71 ± 2.15%	0.79 ± 0.02	**82.84** ± **0.85%**	79.06 ± 0.23%
1 + 2 + 3 + 4 + 5 + 6 + 7	83.54 ± 0.82%	0.79 ± 0.03	81.97 ± 1.38%	79.15 ± 0.56%

All bold values represent the maximum value of each model performance evaluation metric. ACC = classification accuracy, AUC = area under the curve, SEN = sensitivity, SPE = specificity.

**Figure 4.**
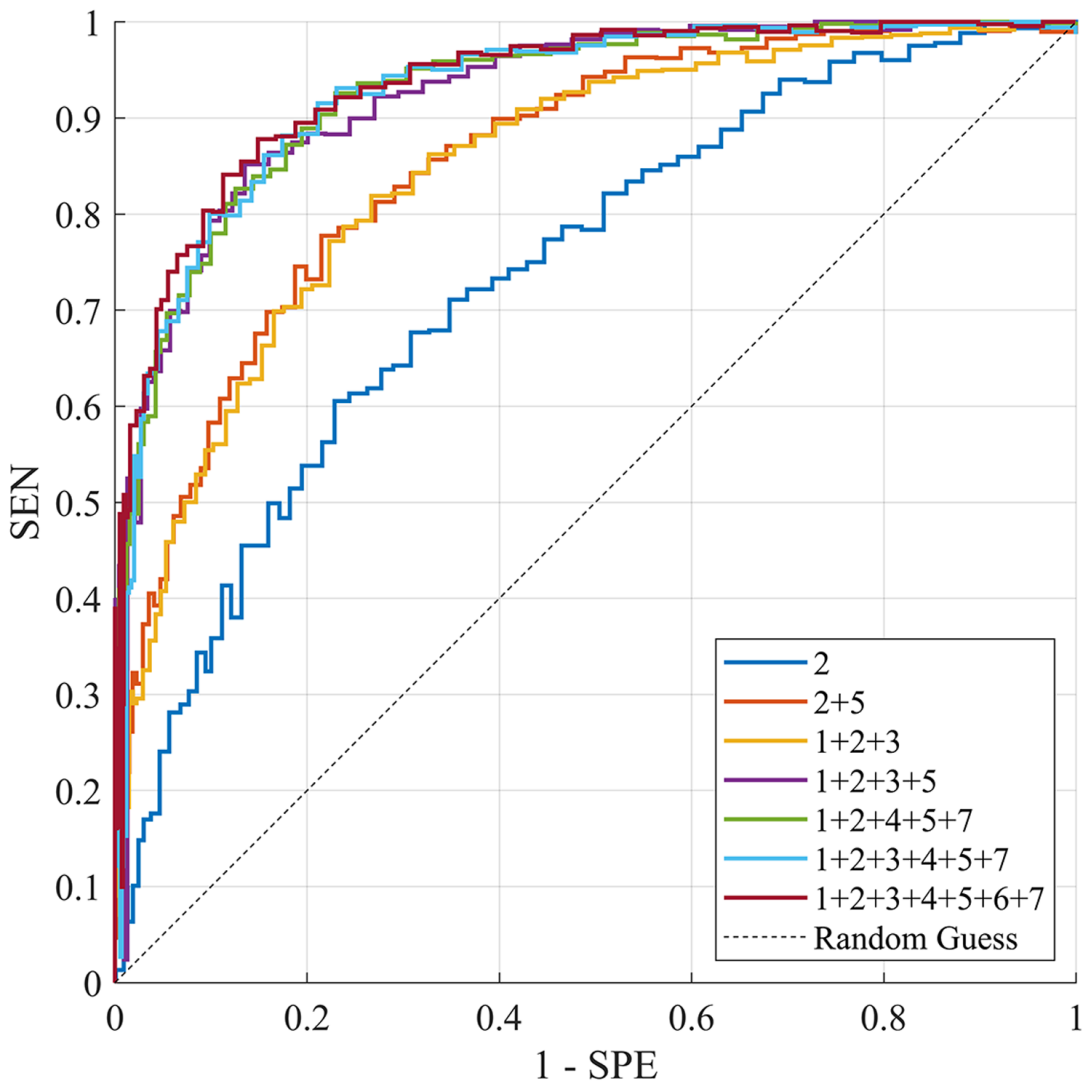
ROC curves of different feature combinations. Maximum tumor diameter in the sagittal plane, ADC value, number of lymph nodes, maximum lymph node diameter, N stage, EMVI, and CRM. These were designated as feature 1, 2, 3, 4, 5, 6, and 7, respectively. The character bar at the lower right of the figure represents models with different combinations of features. The AUC of feature 2 is 0.63 ± 0.04%, the AUC of feature 2 + 5 is 0.78 ± 0.02%, the AUC of feature 1 + 2 + 3 is 0.79 ± 0.03%, the AUC of feature 1 + 2 + 3 + 5 is 0.81 ± 0.01%, the AUC of feature 1 + 2 + 4 + 5 + 7 is 0.85 ± 0.03%, the AUC of feature 1 + 2 + 3 + 4 + 5 + 7 is 0.79 ± 0.02%, the AUC of feature 1 + 2 + 3 + 4 + 5 + 6 + 7 is 0.79 ± 0.03%. ADC = apparent diffusion coefficient, AUC = area under the curve, CRM = circumferential resection margin, EMVI = extramural vascular invasion.

The ROC curves of different feature combinations are shown in Figure 4.

**Figure 5.**
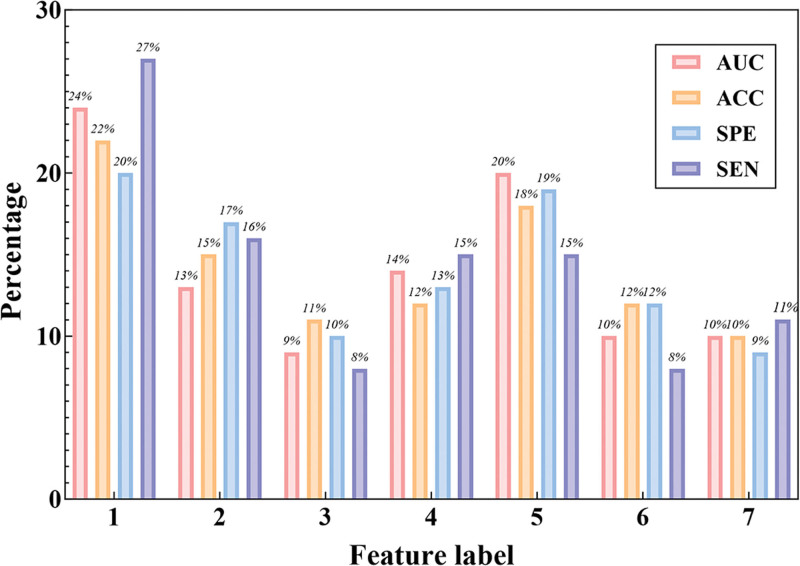
Percentage of features. Among the 127 experiments, the proportions of the maximum sagittal diameter of the tumor, ADC value, number of lymph nodes, maximum diameter of lymph nodes, N stage, EMVI, and CRM in the feature combinations were analyzed and illustrated for the top 50 experiments ranked by ACC, AUC, SEN, and SPE in descending order, where 1–7 represent the feature labels. ACC = classification accuracy, ADC = apparent diffusion coefficient, AUC = area under the curve, CRM = circumferential resection margin, EMVI = extramural vascular invasion, SEN = sensitivity, SPE = specificity.

Among the 127 experiments, the proportions of the maximum sagittal diameter of the tumor, ADC value, number of lymph nodes, maximum diameter of lymph nodes, N stage, EMVI, and CRM in the feature combinations were analyzed and illustrated in Figure 5 for the top 50 experiments ranked by ACC, AUC, SEN, and SPE in descending order, where 1 to 7 represent the feature labels. The research findings indicated that the 3 features: the maximum sagittal diameter of the tumor, ADC value, and N stage, accounted for relatively high proportions in the feature combinations within the top 50 experiments based on predictive performance.

## 5. Discussion

After receiving neoadjuvant therapy, 70 to 80% of rectal cancer patients experience tumor reduction or downward migration, with some patients even experiencing complete tumor regression.^[25]^ According to literature reports,^[26]^ approximately 20% of patients show no residual viable tumor cells following neoadjuvant therapy and surgery. These rectal cancer patients who achieve pathological complete response generally have favorable long-term prognosis, characterized by good local control rates and extended disease-free survival. However, 7 to 37% of patients fail to achieve complete response after treatment.^[27]^ Therefore, pretreatment prediction can greatly assist clinicians in assessing the therapeutic effects of neoadjuvant therapy. Meanwhile, studying the risk factors that influence treatment response and prognosis can help clinicians adjust treatment strategies, thereby improving patient prognosis.

The aim of this study was to explore the potential application of a predictive model based on multiparametric MRI imaging biomarkers in predicting and evaluating the efficacy of neoadjuvant therapy for LARC. A previous study developed a diagnostic model to predict pathological complete response (pCR) after NCRT in LARC patients.^[28]^ The model was built using a set of clinical MRI parameters, including the cylindrical approximated tumor volume, the relative signal intensity of the tumor, and the corresponding reduction rates (RR). It demonstrated relatively good predictive performance. However, it’s worth noting that ADC values, which are widely recognized as significant evaluation indices in the field of oncological imaging, were not included in this previous study. ADC values offer unique information about tissue cellularity and microstructure, which can be crucial for predicting treatment response.

In contrast, by conducting an in-depth analysis of the clinical and imaging data of LARC patients, we obtained a series of meaningful findings in our study. We observed that MRI parameters such as the longest sagittal diameter of the tumor, ADC value, number and maximum diameter of lymph nodes, N stage, EMVI, and CRM before and after neoadjuvant therapy may serve as potential imaging biomarkers for effectively assessing the efficacy of neoadjuvant therapy in rectal cancer.

Our model’s parameters, including the tumor’s longest sagittal diameter (MRI-measurable, showing regression), ADC value (reflecting tissue and tumor response), lymph node count and max diameter (affecting treatment), N stage (linked to aggressiveness), EMVI (predicting metastasis), and CRM (influencing post-op care), are clinically significant.

Relying only on individual parameter assessments for treatment responses has issues. Doctors may differ in interpreting the same parameter changes due to experience gaps, leading to inconsistent advice. Also, these parameters are easily affected by measurement errors and tumor complexity, making individual assessments less objective.

Despite using individually assessed parameters, our model stands out. It provides a more objective and standardized assessment by integrating multiple parameters with advanced algorithms, reducing subjectivity. It can uncover complex parameter-response links that individual assessments may miss, offering a comprehensive patient view. Moreover, it enables personalized treatment planning by considering each patient’s unique parameters, optimizing neoadjuvant therapy and improving outcomes while minimizing complications.

In this study, we developed and validated a clinical prediction model based on multiparametric MRI to effectively predict the therapeutic response of rectal cancer patients undergoing neoadjuvant therapy. During the construction of the prediction model, we utilized an attention mechanism for feature fusion and built an SVM model. Through leave-one-out cross-validation and ablation experiments, we found that the model achieved optimal prediction performance when the feature combination included the longest sagittal diameter of the tumor, ADC value, maximum diameter of lymph nodes, N stage, and CRM. This combination not only exhibited high ACC and AUC but also demonstrated good SEN and SPE, with values of 87.53%, 0.85, 81.26%, and 79.32%, respectively. This finding provides us with a novel method for predicting the efficacy of neoadjuvant therapy in rectal cancer based on multiparametric MRI, which has the potential to serve as an important reference for patient prognosis assessment and treatment strategy determination.

It is noteworthy that in the top 50 experiments ranked by prediction performance, the maximum sagittal diameter of the tumor (Feature 1) exhibited a relatively high proportion across all 4 evaluation metrics, particularly in AUC and SPE, where it accounted for 24% and 27%, respectively. This suggests that the length of the tumor is a highly significant predictor and holds considerable value in assessing the efficacy of neoadjuvant therapy for rectal cancer. The ADC value (Feature 2) maintained a relatively stable proportion across all 4 evaluation metrics, albeit at a lower level. However, it still occupied a certain position in the predictive model, especially in SEN and SPE, where it accounted for 17% and 16%, respectively. As an indicator reflecting changes in tissue microstructure, the ADC value may have certain implications for assessing tumor responsiveness and prognosis. The number of lymph nodes (Feature 3) and the maximum diameter of lymph nodes (Feature 4) exhibited similar proportions across the 4 evaluation metrics, with the maximum diameter of lymph nodes showing a slightly higher proportion, particularly in AUC and SPE, where it accounted for 14% and 15%, respectively. This indicates that the status of lymph nodes is a significant parameter in assessing the efficacy of neoadjuvant therapy for rectal cancer. The N stage (Feature 5) demonstrated relatively high proportions across all 4 evaluation metrics, particularly in AUC and SEN, where it accounted for 20% and 19%, respectively. This underscores the N stage as a pivotal factor in predicting the outcome of neoadjuvant therapy for rectal cancer, closely related to tumor aggressiveness and prognosis. EMVI (Feature 6) showed relatively lower proportions across the 4 evaluation metrics, yet it still held a certain position in the predictive model, particularly in ACC, where it accounted for 12%. As an indicator of vascular invasion in rectal cancer, EMVI may have implications for predicting tumor metastasis risk and prognosis. CRM (Feature 7) demonstrated relatively lower proportions across the 4 evaluation metrics, but it accounted for 11% in SPE, indicating its role in the predictive model. As an indicator of surgical quality, CRM may provide valuable insights into predicting the efficacy of neoadjuvant therapy for rectal cancer and patient prognosis. The longest sagittal diameter of the tumor, ADC value, and N stage occupied a relatively high proportion in the feature combinations. This result further underscores the importance of these 3 parameters in predicting the efficacy of neoadjuvant therapy for rectal cancer. The longest sagittal diameter of the tumor, as a direct indicator reflecting tumor size, can intuitively demonstrate tumor regression.^[29]^ The ADC value, serving as a sensitive indicator of microstructural changes in tissue, can reveal the diffusion of water molecules within the tumor, thereby indirectly reflecting tumor responsiveness.^[30]^ Moreover, N stage, an important parameter for assessing tumor aggressiveness and prognosis, also holds significance in predicting therapeutic efficacy.^[31]^ Additionally, although the maximum diameter of lymph nodes and CRM played a role in the prediction model, their proportions were relatively lower in the top 50 experiments. This may be related to factors such as sample size, data distribution, and algorithms used in different experiments. However, this does not imply that these 2 parameters lack value in predicting the efficacy of neoadjuvant therapy for rectal cancer. On the contrary, they may still serve as important auxiliary parameters, providing strong support for model optimization and the formulation of personalized treatment plans.

This study has contributed valuable insights, yet several limitations warrant careful consideration, along with corresponding strategies we plan to adopt for future improvement. Firstly, post-treatment inflammatory edema and fibrosis may lead to staging inaccuracies. To address this, future research will incorporate advanced imaging techniques like PET-CT to better distinguish true disease progression from treatment-related changes, improving staging precision. Secondly, the relatively small and single-center sample size limits statistical power and generalizability. We plan to conduct multi-center studies with larger, more diverse cohorts to enhance result robustness and applicability across varied clinical settings in the future. Thirdly, the lack of standardized assessment methods currently hampers result comparability. In future studies, we will stay abreast of the latest developments in assessment methodologies, continuously monitoring and integrating updated, universally recognized standards into our research. Fourthly, the current follow-up duration may not fully capture long-term treatment effects. Future studies will implement extended follow-up protocols, with regular assessments over several years, to monitor outcomes more comprehensively and identify late-occurring effects. Finally, we are aware that the risk of overfitting and the absence of independent external validation may cast a slight shadow on the complete confidence in our current findings. To gently allay these concerns, future analyses will incorporate cross-validation techniques. Additionally, we will actively seek opportunities for independent validation through collaborations with other research teams or by engaging external review panels. Through these measures, we hope to gradually strengthen the credibility and reliability of our research conclusions.

## 6. Conclusions

In summary, this study has developed a model using multiparametric MRI imaging indicators to predict the efficacy of neoadjuvant therapy for rectal cancer. This model demonstrates high predictive accuracy and clinical application value, providing important insights for patient prognosis assessment and surgical plan adjustment. In the future, we will continue to delve into the application of multiparametric MRI imaging indicators in the diagnosis and treatment of rectal cancer, aiming to offer more precise and personalized treatment plans for patients. Additionally, we will keep abreast of the development of new technologies and methodologies to continuously enhance the level of diagnosis and treatment for rectal cancer, ultimately bringing better therapeutic outcomes and improved quality of life to patients. In the future, we plan to adapt the prediction model into a user-friendly clinical mini-program that will allow physicians to enter relevant parameters and swiftly generate personalized treatment outcome predictions for patients.

## Author contributions

**Data Curation**: Xinying Wu.

**Methodology**: Jing Cheng, Xinying Wu.

**Writing – original draft**: Jing Cheng.

**Writing – review & editing**: Xinying Wu.
